# A slow-forming isopeptide bond in the structure of the major pilin SpaD from *Corynebacterium diphtheriae* has implications for pilus assembly

**DOI:** 10.1107/S1399004714001400

**Published:** 2014-04-26

**Authors:** Hae Joo Kang, Neil G. Paterson, Chae Un Kim, Martin Middleditch, Chungyu Chang, Hung Ton-That, Edward N. Baker

**Affiliations:** a Maurice Wilkins Centre for Molecular Biodiscovery and School of Biological Sciences, University of Auckland, Private Bag 92019, Auckland 1142, New Zealand; b Cornell High Energy Synchrotron Source and Macromolecular Diffraction Facility at CHESS (MacCHESS), Cornell University, Ithaca, NY 14853, USA; c Department of Microbiology and Molecular Genetics, University of Texas–Houston Medical School, Houston, TX 77030, USA

**Keywords:** Gram-positive bacterial pili, pilus assembly, isopeptide bond

## Abstract

The Gram-positive organism *Corynebacterium diphtheriae*, the cause of diphtheria in humans, expresses pili on its surface which it uses for adhesion and colonization of its host. These pili are covalent protein polymers composed of three types of pilin subunit that are assembled by specific sortase enzymes. A structural analysis of the major pilin SpaD, which forms the polymeric backbone of one of the three types of pilus expressed by *C. diphtheriae*, is reported. Mass-spectral and crystallographic analysis shows that SpaD contains three internal Lys–Asn isopeptide bonds. One of these, shown by mass spectrometry to be located in the N-terminal D1 domain of the protein, only forms slowly, implying an energy barrier to bond formation. Two crystal structures, of the full-length three-domain protein at 2.5 Å resolution and of a two-domain (D2-D3) construct at 1.87 Å resolution, show that each of the three Ig-like domains contains a single Lys–Asn isopeptide-bond cross-link, assumed to give mechanical stability as in other such pili. Additional stabilizing features include a disulfide bond in the D3 domain and a calcium-binding loop in D2. The N-terminal D1 domain is more flexible than the others and, by analogy with other major pilins of this type, the slow formation of its isopeptide bond can be attributed to its location adjacent to the lysine used in sortase-mediated polymerization during pilus assembly.

## Abstract 2

Two crystal structures of the major pilin SpaD from *C. diphtheriae* have been determined at 1.87 and 2.5 Å resolution. The N-terminal domain is found to contain an isopeptide bond that forms slowly over time in the recombinant protein. Given its structural context, this provides insight into the relationship between internal isopeptide-bond formation and pilus assembly.

## 1. Introduction   

Gram-positive pathogenic bacteria produce a range of cell surface-associated virulence factors that promote colonization, invasion, infection and mediation of the host immune response. One of the most prominent of these is the bacterial pilus, a long, adhesin-tipped protein filament that extends several micrometres from the cell and aids colonization in motile environments (Proft & Baker, 2009 ▶).

Gram-positive pili are assembled as a remarkable type of covalent protein polymer. Formed from a single chain of covalently linked subunit proteins (pilins), they comprise an adhesin subunit located at the tip, referred to as a minor pilin, followed by many copies of a major pilin that forms the polymeric shaft and finally a second minor pilin that terminates pilus extension and mediates cell-wall anchoring at the base (Telford *et al.*, 2006 ▶; Mandlik *et al.*, 2008 ▶; Hendrickx *et al.*, 2011 ▶). The subunits are covalently linked *via* sortase-catalysed isopeptide bonds. The sortase enzyme recognizes a characteristic sequence motif (LP*X*TG, or variants depending on the specific sortase involved) near the C-terminus of the pilin subunit, cleaves this motif between Thr and Gly, and joins the new Thr carboxyl to the ∊-amino group of a conserved lysine in the N-terminal domain of the next subunit (Ton-That *et al.*, 2004 ▶; Kang *et al.*, 2007 ▶; El Mortaji *et al.*, 2012 ▶). Following assembly of the pilus shaft and capping with the basal minor pilin, the whole assembly is covalently linked to the bacterial cell wall by a housekeeping sortase (Marraffini *et al.*, 2004 ▶; Swaminathan *et al.*, 2007 ▶).

Pili from the Gram-positive organism *Corynebacterium diphtheriae* were the first such pili to be characterized in molecular terms, some ten years ago. This organism produces three distinct pilus assemblies (Ton-That *et al.*, 2004 ▶; Ton-That & Schneewind, 2003 ▶), hereinafter referred to by their major pilins as SpaA, SpaD and SpaH pili. An individual gene cluster encodes each assembly, containing the major pilin, two minor pilins and the associated sortases required for polymerization (Ton-That *et al.*, 2004 ▶). The three pili have different host cell ligands for their adhesins, with SpaA pili preferring pharyngeal epithelial cells and SpaD and SpaH pili showing stronger adhesion to laryngeal and lung epithelial cells (Mandlik *et al.*, 2007 ▶).

The modular nature of pilus formation is replicated in the architecture of individual pilin subunits. Crystal structures of major pilins from *Streptococcus pyogenes* (Kang *et al.*, 2007 ▶), *S. agalactiae* (Krishnan *et al.*, 2007 ▶), *C. diphtheriae* (Kang, Paterson, Gaspar *et al.*, 2009 ▶), *S. pneumoniae* (Spraggon *et al.*, 2010 ▶; Gentile *et al.*, 2011 ▶; Paterson & Baker, 2011 ▶) and *Bacillus cereus* (Budzik *et al.*, 2009 ▶) reveal multiple immunoglobulin (Ig)-like domains: two in the case of *S. pyogenes* Spy0128, three for *S. agalactiae* GBS80 and *C. diphtheriae* SpaA, and four for *B. cereus* BcpA and *S. pneumoniae* RrgB. Similar modular domains are also used in the basal pilins and the C-­terminal portions of the adhesins that connect to the polymeric pilus stalk (Krishnan *et al.*, 2007 ▶; Izoré *et al.*, 2010 ▶; Linke *et al.*, 2010 ▶; Pointon *et al.*, 2010 ▶).

A characteristic feature of these modular domains is the presence of internal isopeptide bonds, *i.e.* amide bonds formed between Lys and Asn (or Asp) side chains that form internal cross-links within each domain (Kang & Baker, 2011 ▶) and provide exceptional resistance to tensile stress and to proteolytic and thermal denaturation (Alegre-Cebollada *et al.*, 2010 ▶; Kang & Baker, 2009 ▶). Isopeptide-bond formation is autocatalytic, and is dependent on a neighbouring acidic residue (Asp or Glu) and p*K*_a_ changes that result from the hydrophobic internal environment (Kang *et al.*, 2007 ▶). These bonds are assumed to form when the pilin subunits fold and the three residues involved are brought into close proximity. However, it appears that in some cases the isopeptide bond does not form until the pilin subunit is stabilized either by incorporation into the pilus or by docking during sortase-mediated polymerization. Thus, the N-terminal domains of *B. cereus* BcpA and *S. pneumoniae* RrgB have the requisite Lys, Asn and Glu residues but only form internal isopeptide bonds during pilus assembly, not in the recombinant proteins (Paterson & Baker, 2011 ▶; Budzik *et al.*, 2009 ▶). Studies on RrgB suggest that this is owing to the fact that the isopeptide bond-forming Asn residue is adjacent within the conserved YPKN pilin motif to the essential Lys involved in polymerization, and an energy barrier must be overcome before the Asn can be brought into close proximity to the other isopeptide-forming residues (El Mortaji *et al.*, 2012 ▶; Paterson & Baker, 2011 ▶).

In this study, we present two crystal structures of the major pilin SpaD from *C. diphtheriae* refined at 1.87 and 2.5 Å resolution, showing a related phenomenon. An intact isopeptide bond is found in the N-terminal domain, but mass-spectrometric data show that this bond forms slowly over time in the recombinant protein. The position of this bond in relation to the site at which the intermolecular linkage is formed, together with data from other pilin structures, provides insight into the relationship between internal isopeptide-bond formation and pilus assembly.

## 2. Methods   

### 2.1. Cloning and protein expression   

The DNA sequence of SpaD (gi:38232859) was amplified from *C. diphtheriae* strain NCTC13129 genomic DNA and cloned using Gateway cloning methods (Moreland *et al.*, 2005 ▶). The following primers were used for the first round of PCR for the full-length mature form of SpaD containing residues 27–455: forward, 5′-TTCCAAGGTCCG**GGTG** **CCGTCGCTATTGCA**-3′; reverse, 5′-GAAAGCTGGGTGCTA**GGTGCCCTGCTTGATGTTTTTA**-3′ (gene-specific sequences are shown in bold). For the SpaD D1 construct encoding residues 49–183, the following primers were used: forward, 5′-TTCCAAGGTCCG**GAACGAAAGGGCTCGCTGA**-3′; reverse, 5′-GAAAGCTGGGTGCTA**GGTTTCGGTGTTCTTCGG**-3′. Another round of PCR was performed using the generic primers containing *attB* sequences for Gateway cloning and the sequence encoding an N-terminal, human rhinoviral (HRV) 3C protease site: forward, 5′-GGGGACAAGTTTGTACAAAAAAGCAGGCTCTCTGC­AGGTACTCTTCCAAGGTCCG-3′; reverse, 5′-GGGGA­CCACTTTGTACAAGAAAGCTGGGTG-3′. The final PCR product was first cloned into the entry vector pDONR221 (Invitrogen) *via* a BP reaction, and an LR reaction was then used to clone into the expression vector pDEST17 (Invitrogen) for expression of a His-tagged construct. The final constructs were transformed into *Escherichia coli* BL21 (DE3) cells. The full-length native SpaD was overexpressed in ZYP-5052 autoinduction media (Studier, 2005 ▶) at 37°C for the first 4 h, followed by transfer to 18°C for a further 24 h. SeMet SpaD was expressed in the same manner as the native SpaD using PASM-5052 medium (Studier, 2005 ▶). The expression of the SpaD D1 construct was carried out in Magnificent Broth (MacConnell Research) at 37°C for 4 h, followed by induction with 1 m*M* isopropyl β-d-thiogalactopyranoside (IPTG) and incubation at 18°C for a further 60 h. All of the culture medium were supplemented with ampicillin (100 µg ml^−1^) and chloramphenicol (34 µg ml^−1^).

### 2.2. Protein purification   

The SpaD proteins were purified using similar procedures to those used for the purification of SpaA (Kang, Paterson & Baker, 2009 ▶). Briefly, cleared cell lysate was loaded onto a 5 ml HisTrap column (GE Healthcare) charged with Ni^2+^, and the His-tagged SpaD proteins were eluted with a gradient of 20–500 m*M* imidazole. To remove the His tag, the eluted proteins were mixed with human rhinovirus (HRV) 3C protease (1 mg per ∼50 mg SpaD), 10 m*M* DTT and 2 m*M* EDTA and dialyzed against 50 m*M* Tris–HCl pH 8.0, 300 m*M* NaCl overnight at 277 K. For SeMet-substituted SpaD, the buffers were supplemented with 0.4 m*M* DTT from this step to prevent selenomethionine oxidation. The dialyzed sample was passed through a charged HisTrap column to collect the flowthrough containing untagged SpaD. Further purification was carried out using size-exclusion chromatography (SEC) on a Superdex 75 10/300 column (GE Healthcare) with SEC buffer (10 m*M* Tris–HCl pH 8.0, 50 m*M* NaCl). The final product contained two additional N-terminal residues, Gly and Pro, after His-tag removal.

### 2.3. Crystallization, high-pressure cryocooling and data collection for SpaD   

SpaD was initially crystallized by vapour diffusion using sitting drops in 96-well Intelli-Plates (Hampton Research) at 291 K set up with a Cartesian Honeybee dispensing system (Genomic Solutions). After optimization, full-length native SpaD protein was crystallized at a protein concentration of 77 mg ml^−1^ in SEC buffer with reservoir solution consisting of 15% PEG 600, 0.2 *M* imidazole–malate pH 5.5. Full-length SeMet SpaD crystals were obtained from a mother liquor consisting of 0.1 *M* imidazole–HCl pH 8.0, 30% 2-methyl-2,4-pentanediol (MPD), 10% PEG 4000. Crystals started to appear after 1–2 weeks in 2 µl sitting drops consisting of a 1:1 mixture of protein solution and reservoir solution at 18°C. When cryocooled in liquid nitrogen using standard cryoprotection protocols (reservoir solutions containing either 25% ethylene glycol or glycerol for native SpaD and 40% MPD for the SeMet protein), these crystals showed severe anisotropy, diffracting to 3 Å in one direction but typically to 8 Å in the other. The Bragg spots were also too close together to be resolved owing to high mosaicity and a long unit-cell edge.

In order to improve the data quality, the crystals were cryocooled under high pressure at the Cornell High Energy Synchrotron Source (CHESS; Kim *et al.*, 2005 ▶) prior to data collection. Purified SpaD protein was transported to CHESS and crystallized on site as described above. The SpaD crystals were then coated with NVH oil (Hampton Research) to prevent crystal dehydration, loaded into the high-pressure cryocooling apparatus and pressurized in helium gas to 200 MPa. After 5 min, the crystals were cryocooled to liquid-nitrogen temperature while still under high pressure. A minute later, the helium pressure was released and the crystals were subsequently handled at low temperature and ambient pressure for cryo-crystallographic data collection.

Diffraction data were collected from the high-pressure cryocooled SpaD crystals on beamline F1 (100 µm beam diameter, ADSC Quantum 270 CCD detector, X-ray wavelength of 0.9170 Å) at the Cornell High Energy Synchrotron Source (CHESS). All data were collected at 90 K (N_2_ gas stream) and ambient pressure with an oscillation angle of 0.5° per image. Bragg diffraction spots were visible up to 2.1 Å, but anisotropic diffraction was observed beyond 2.8 Å. Therefore, the data set was cut off at 2.5 Å for structure determination and refinement.

### 2.4. Crystallization and data collection for SeMet SpaD_tryp_   

In order to identify a stable fragment of SpaD, limited proteolysis was performed by adding trypsin (Promega) to full-length SeMet-substituted SpaD in a 1:300(*w*:*w*) ratio and incubating at 37°C for up to 20 h. Analysis by SDS–PAGE revealed a single strong band corresponding to a molecular weight smaller than the full-length SpaD. This was shown by mass spectrometry of the digested products to be a species with a molecular mass of 30 456.00 Da. This closely matches the theoretical mass (molecular mass of 30 457.10 Da) of residues 180–455 of SeMet SpaD, after accounting for the loss of 34 Da from two isopeptide bonds. For crystallization of this species, SeMet SpaD_tryp_, 1 ml of 7.0 mg ml^−1^ SeMet SpaD in SEC buffer was digested with 60 ng trypsin for 20 h, followed by concentration to 40 mg ml^−1^ using a 10 kDa molecular-weight cutoff concentrator. No further purification was carried out. Crystallization was then performed with the Cartesian dispensing robot using sitting drops comprising 100 nl protein solution and 100 nl reservoir solution (47% MPD and 2% *t*-­butanol) at 18°C. The crystals were cryocooled by plunging them into liquid nitrogen without further cryoprotection and proved to be of high quality, diffracting to better than 2.0 Å resolution. An initial data set was collected in-house using a Rigaku MicroMax-007 HF generator with a rotating Cu anode (Rigaku) and a Mar345dtb image plate (MAR Research), covering a total rotation range of 200° with 0.5° oscillation. Following analysis, a second data set was collected using 1° oscillation images, covering a total oscillation of 864°.

### 2.5. Structure solution   

Data sets collected from SeMet SpaD_tryp_ were processed separately using *XDS* (Kabsch, 2010 ▶) and combined prior to scaling with *SCALA* (Winn *et al.*, 2011 ▶). Bijvoet pairs were treated as non-equivalent during scaling. The crystals were found to belong to space group *P*2_1_2_1_2_1_, with unit-cell parameters *a* = 35.99, *b* = 81.33, *c* = 92.37 Å, α = β = γ = 90.0° and one molecule in the asymmetric unit (*V*_M_ = 2.27 Å^3^ Da^−1^, 45.8% solvent content). *autoSHARP* (Vonrhein *et al.*, 2007 ▶) and *SHELX* (Schneider & Sheldrick, 2002 ▶) were used to find four anomalous sites that were manually assigned to two S atoms, one Ca atom and one Se atom based on the crystal structure of *C. diphtheriae* SpaA (Kang, Paterson, Gaspar *et al.*, 2009 ▶). Sites were refined and phases were calculated using *SHARP* (Vonrhein *et al.*, 2007 ▶) followed by density modification with *DM* (Winn *et al.*, 2011 ▶). Model building with *ARP*/*wARP* (Langer *et al.*, 2008 ▶) led to the building of 227 residues, with subsequent cycles of manual model building in *Coot* (Emsley *et al.*, 2010 ▶) and refinement using *BUSTER* (Blanc *et al.*, 2004 ▶) extending the model to 272 consecutive residues. After addition of water molecules using the Find Waters feature in *Coot*, and a single Ca^2+^ ion (identified from its anomalous density and coordination environment), final values of *R*_work_ = 0.157 and *R*_free_ = 0.190 (5.1% of the reflections were selected randomly for the *R*_free_ calculation) were obtained. The final model of SeMet SpaD_tryp_ comprised residues 184–455 of SeMet SpaD, which is consistent with the MS analysis of the digested product.

Native full-length SpaD diffraction data from the high-pressure crystals were processed using *XDS* (Kabsch, 2010 ▶) and scaled with *SCALA* (Winn *et al.*, 2011 ▶). These data were distinctly anisotropic, as noted above, and the data set used for structure solution and refinement was cut off at a resolution of 2.5 Å, where the merging *R* factor was 0.70. The crystals were found to belong to space group *P*2_1_2_1_2_1_, with unit-cell parameters *a* = 32.83, *b* = 56.79, *c* = 435.84 Å, α = β = γ = 90.0° and two molecules in the asymmetric unit (*V*_M_ = 2.17 Å^3^ Da^−1^, 43.3% solvent content). The structure was solved by molecular replacement with *Phaser* (McCoy *et al.*, 2007 ▶), using the two-domain SeMet SpaD_tryp_ structure as a search model. Two copies could be placed in the asymmetric unit. Cycles of manual building with *Coot* (Emsley *et al.*, 2010 ▶) and refinement using *BUSTER* (Blanc *et al.*, 2004 ▶) with TLS restraints (Winn *et al.*, 2001 ▶) enabled the addition of residues 42–184 from the missing D1 domain to the model, although several loop regions were disordered. The final model comprised two molecules of full-length SpaD, comprising residues 42–436 and 440–455 for chain *A* and residues 42–62, 77–212, 215–434 and 436–453 for chain *B*. This model had final values of *R*_work_ = 0.187 and *R*_free_ = 0.250 (5.0% of the reflections were selected randomly for the *R*_free_ calculation). Validation with *MolProbity* (Chen *et al.*, 2010 ▶) showed 97.1% of residues in the most favoured regions of the Ramachandran plot. Full details of data-collection, phasing and refinement statistics for both SeMet SpaD_tryp_ and full-length native SpaD are given in Table 1 ▶. For structural comparisons, the *DALI* server (http://ekhidna.biocenter.helsinki.fi/dali_server/start) was used.

### 2.6. Mass-spectral analyses   

The molecular masses of the intact proteins in solution were determined by infusion ESI-TOF mass spectrometry (MS) on a Q-STAR XL Hybrid MS/MS system (Applied Biosystems) in 50% acetonitrile and 0.1% formic acid. The raw MS data were deconvoluted using the Bayesian Protein Reconstruct tool in the *BioAnalyst* software (Applied Biosystems). For the MS analysis of protein crystals, the crystals were washed briefly in acetonitrile several times and then dissolved in deionized water. For identifying isopeptide bond-containing peptides, samples of purified SpaD pili or recombinant SpaD protein were digested and analysed in a similar way to that previously described for SpaA (Kang, Paterson, Gaspar *et al.*, 2009 ▶). Briefly, SDS–PAGE gel bands containing ∼10 µg of SpaD or SpaD pili were diced and washed with 50% acetonitrile and 25 m*M* NH_4_HCO_3_, followed by incubation in 100% acetonitrile and drying under vacuum. The dried gel pieces were incubated at 37°C with the proteases trypsin (Promega) and AspN endopeptidase (Roche) dissolved in 25 m*M* NH_4_HCO_3_ and 10% acetonitrile. The digested samples were analyzed using a Q-STAR XL Hybrid MS/MS system (Applied Biosystems) as described in Kang, Paterson, Gaspar *et al.* (2009 ▶).

## 3. Results   

### 3.1. Structure determination of SpaD   

*C. diphtheriae* SpaD is a 490-residue protein containing a predicted N-terminal signal peptide of 32 residues and a sortase-recognition motif LPMTG encompassing residues 458–462. Post-expression processing yields a 429-residue mature protein comprising residues 33–461. After testing several different constructs of SpaD, we were able to obtain diffraction-quality crystals from a construct containing residues 27–455, comprising most of the mature full-length SpaD but lacking the C-terminal sortase motif residues. Both native and SeMet-substituted full-length SpaD were purified from *E. coli* and crystallized in space group *P*2_1_2_1_2_1_. Typical X-­ray diffraction at synchrotron light sources was anisotropic with very high mosaicity, hindering data processing and precluding structure determination by SAD/MAD phasing.

We therefore sought to prepare a stable truncated fragment of SpaD that could be crystallized and used to solve the structure of the full-length protein by molecular replacement. This was achieved by partial trypsin digestion of the full-length selenomethionine-labelled form of SpaD (SeMet SpaD). Mass spectrometry confirmed the mass of the resulting product to be 30 456.00 Da, corresponding to residues 180–455. This truncated form of SpaD (SeMet SpaD_tryp_) was crystallized in space group *P*2_1_2_1_2_1_ and its structure was solved by single-wavelength anomalous diffraction (SAD) methods from in-house data collected at a wavelength of 1.5418 Å, utilizing the signals from one Se atom, one Ca^2+^ ion and two S atoms (from a disulfide bond). The model, comprising residues 184–455, was refined using data to 1.87 Å resolution (*R* = 15.7%, *R*_free_ = 19.0%; PDB entry 4hsq).

Improved crystals of the full-length native SpaD were then obtained by cryocooling the crystals under high pressure (Kim *et al.*, 2005 ▶), which enabled data processing to 2.5 Å resolution (Table 1 ▶). The structure was solved by molecular replacement using the structure of the truncated form (SeMet SpaD_tryp_) as a search model. This full-length structure, which contains two SpaD molecules in the asymmetric unit, was refined at 2.5 Å resolution (*R* = 18.7%, *R*_free_ = 25.0%; PDB entry 4hss). In the final model, one protomer is complete apart from residues 27–41 (the N-terminus) and 437–439, whereas in the other a large loop between residues 63 and 76, smaller loops between residues 212 and 214 and residues 435 and 436, and the C-­terminal residues 454–455 could not be modelled owing to insufficient density. Full refinement statistics are given in Table 1 ▶.

### 3.2. Overall structure and structural comparisons   

**Figure 1:**
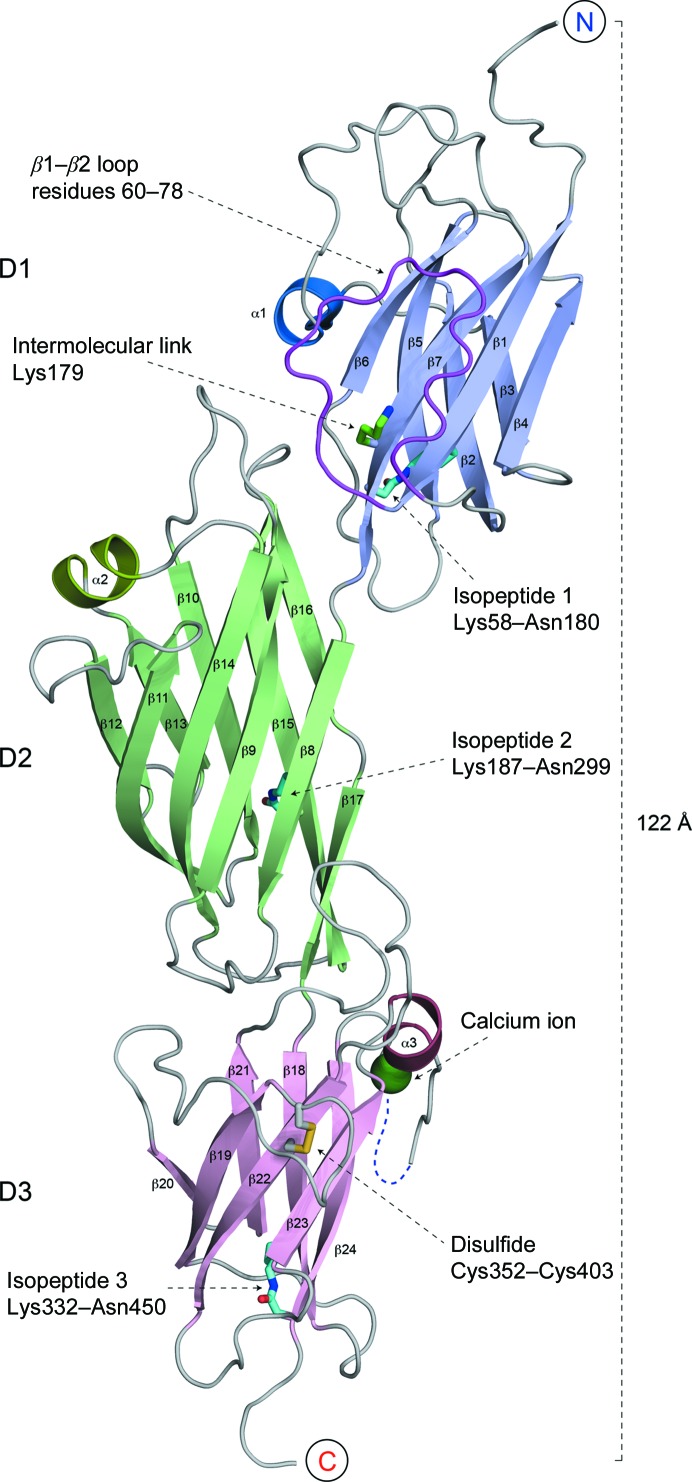
Overall structure of full-length *C. diphtheriae* SpaD. Cartoon diagram showing the three-domain structure comprising D1 (light purple), D2 (green) and D3 (pink). Key features highlighted are the isopeptide bonds (cyan), Ca^2+^-binding site (green sphere), S—S bond (yellow), β1–β2 loop (magenta) and Lys179 (green).

SpaD is an elongated molecule of ∼125 Å in length built from three tandem domains with Ig-type folds (Fig. 1 ▶). As in some of the other Gram-positive major pilins, these three domains comprise a single CnaA-type domain flanked by two CnaB-type domains. The two CnaB-type domains, D1 (residues 42–181) and D3 (residues 325–455), have a seven-stranded β-sandwich fold with reverse-Ig topology characteristic of CnaB domains (Deivanayagam *et al.*, 2000 ▶), and have a root-mean-square difference (r.m.s.d.) of 1.8 Å over 92 equivalent C^α^ atoms with a sequence identity of 28%. The D2 domain (residues 182–324) has the typical CnaA fold (Deivanayagam *et al.*, 2002 ▶), in which nine strands form a partially open β-barrel. Like many other CnaA-type and CnaB-type domains (Kang & Baker, 2011 ▶), those in SpaD also contain intramolecular isopeptide bonds, which are described in the following sections.

The principal axis of the N-terminal D1 domain is angularly disposed with respect to that of the D2 domain that follows, with D1 positioned above the opening of the D2 β-barrel. In contrast, domains D2 and D3 are almost linearly connected, with strands β17 and β18 running through the length of both domains (Fig. 1 ▶). The angular ‘bend’ between the D1 and D2/D3 domains differs in the two molecules of the asymmetric unit (Fig. 4*d*), however. Aside from this, the two copies of SpaD in the asymmetric unit are very similar, with r.m.s.d.s of 0.28 Å between the two D1 domains over 126 C^α^ atoms and of 0.51 Å between the two D2/D3 domain pairs over 264 C^α^ atoms.

The three-domain architecture of SpaD corresponds to that first found in the structure of SpaA, another major pilin of *C. diphtheriae* (Kang, Paterson, Gaspar *et al.*, 2009 ▶). Despite the low sequence identities between them (22–28%), each domain of SpaD closely matches the corresponding domain of SpaA, with an r.m.s.d. of ∼2 Å in each case; the M and C domains of SpaA are the closest structural homologues of the D2 and D3 domains of SpaD, respectively. The r.m.s.d. increases to 4.3 Å when the whole structures are compared, because of the different D1 dispositions relative to the D2/D3 domains (Fig. 4*d*). The major pilin FimP of *Actinomyces oris* (Persson *et al.*, 2012 ▶) is the closet homologue of the full-length SpaD, as well as its D2–D3 structure, with *Z*-scores of 28 and 25, respectively, from a *DALI* search (Holm & Rosenström, 2010 ▶). Close structural homologues of each SpaD domain are also found in other pilins. The closest homologue of D1 is the N-terminal domain of *A. oris* FimP (Persson *et al.*, 2012 ▶), with an r.m.s.d. of 1.7 Å over 132 C^α^ atoms, whereas the N2 domain of *Streptococcus gordonii* adhesin Sgo0707 (Nylander *et al.*, 2013 ▶) is the closet homologue of D2, with an r.m.s.d. of 2.4 Å over 129 C^α^ atoms. The D3 domain has 30% sequence identity and an r.m.s.d. of 1.7 Å over 99 C^α^ atoms with the N2 domain of the *S. agalactiae* minor pilin GBS52, and is similarly related to the CnaB-type domains of *B. cereus* BcpA (Budzik *et al.*, 2009 ▶) and *S. pneumoniae* RrgB (Spraggon *et al.*, 2010 ▶; Gentile *et al.*, 2011 ▶; Paterson & Baker, 2011 ▶). These relationships emphasize the modular nature of the pilin proteins and other adhesins found on the surface of Gram-positive organisms.

**Figure 2:**
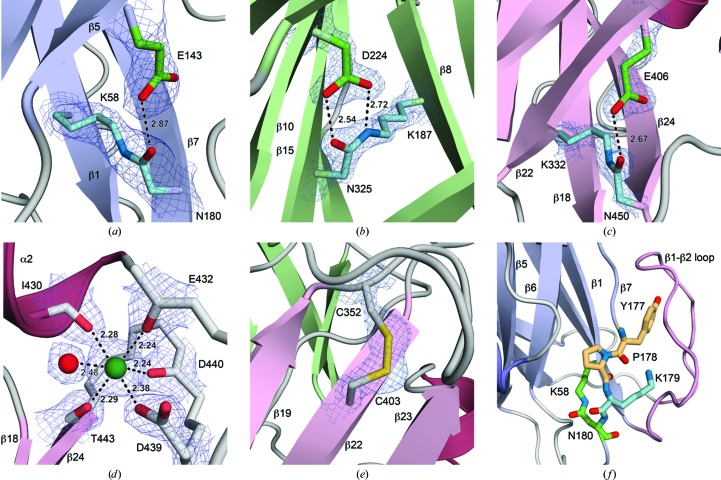
Key features of SpaD, with observed electron density. (*a*) and (*f*) are rendered from the full-length SpaD structure with electron density from a 2*F*_o_ − *F*_c_ electron-density map contoured at 0.20 e Å^−3^ (1.0σ) and (*b*)–(*e*) are rendered from the higher resolution truncated form with electron density from a 2*F*_o_ − *F*_c_ electron-density map contoured at 0.69 e Å^−3^ (2.0σ). Bonding distances (Å) are given where appropriate. (*a*) The D1 isopeptide bond and catalytic glutamic acid, (*b*) the D2 isopeptide bond with catalytic aspartic acid, (*c*) the D3 isopeptide bond with catalytic glutamic acid, (*d*) the calcium ion and coordinating ligands, (*e*) the disulfide bond and (*f*) the YPKN pilin motif, shown in stick form, with the tryptophan and proline residues in yellow, the intermolecular isopeptide-forming lysine Lys179 in cyan and the D1 isopeptide bond in green.

The D3 domain contains a calcium-binding site formed within a loop between strands β23 and β24 (Fig. 1 ▶). Octa­hedral coordination is provided by four main-chain carbonyl O atoms from residues Ile430, Glu432, Asp440 and Thr443; the carboxyl group of Asp439; and an ordered water molecule (Fig. 2 ▶ *d*). The calcium ion was identified by the electron density in conjunction with an average metal–ligand bond length of 2.27 Å for the main-chain carbonyls and 2.43 Å for the water and carboxyl group. The calcium ion was also used during phasing of SeMet SpaD_tryp_ and gave rise to an anomalous signal consistent with that expected from calcium at an X-ray wavelength of 1.5418 Å. Although SpaA also contains a calcium-binding site, location is different from that in SpaD. The calcium site in SpaA (Kang, Paterson, Gaspar *et al.*, 2009 ▶) is located in the middle domain M (equivalent to D2 in SpaD), and is on the opposite face of the molecule from that in SpaD. Calcium-binding sites are commonly found in other cell-surface adhesins as well, for example the major pilins GBS80, FimA and FimP and the antigen I/II adhesin (Vengadesan *et al.*, 2011 ▶; Mishra *et al.*, 2011 ▶; Persson *et al.*, 2012 ▶; Forsgren *et al.*, 2010 ▶). Although the locations of these binding sites are not conserved and there is no direct evidence that they contribute to stability, their frequent occurrence suggests that they may play a role in enhancing the local stability of surface features on these proteins.

Another potentially stabilizing modification in SpaD is the presence of a disulfide bond between Cys352 and Cys402 in the D3 domain, cross-linking strands β19 and β22 (Figs. 1 ▶ and 2 ▶ *e*). A disulfide bond is found at the analogous position in SpaA, but that in SpaD is fully formed as opposed to the partially formed bond observed in SpaA (Kang, Paterson, Gaspar *et al.*, 2009 ▶).

### 3.3. Intramolecular isopeptide bonds in SpaD   

Interpretation of the electron density showed the clear presence of three isopeptide bonds in the structure of SpaD, cross-linking the first and last β-strands of the CnaB-type domains D1 and D3 and the first and second-last strands of the CnaA-type D2 domain (Fig. 1 ▶). All are Lys–Asn bonds that form autocatalytically with loss of ammonia as described previously (Kang *et al.*, 2007 ▶). The isopeptide bonds link Lys58 and Asn180 in the D1 domain, Lys187 and Asn299 in the D2 domain and Lys332 and Asn450 in the D3 domain with the essential catalytic acids provided by Glu143, Asp224 and Glu406, respectively. The presence of these isopeptide bonds was also confirmed by electrospray ionization time-of-flight (ESI-TOF) mass spectrometry of dissolved crystals, which showed a loss of 17 Da per isopeptide bond owing to the loss of ammonia (see §[Sec sec3.4]3.4 for further details).

Both isopeptide-bond stereoisomers are present in SpaD, with the D1 and D3 domain bonds having the *trans* peptide configuration and the bond in the D2 domain having a *cis* stereochemistry. In both *trans* isopeptide bonds the carboxylic group of the catalytic Glu residue adopts a side-on position relative to the Asn side chain, thus forming only a single hydrogen bond, to the isopeptide carbonyl O atom (Figs. 2 ▶ *a* and 2 ▶ *c*). In contrast, the *cis* isopeptide bond of the D2 domain forms two hydrogen bonds through its carbonyl and amido groups to the two carboxyl O atoms of Asp224 (Fig. 2 ▶ *b*).

### 3.4. The slow-forming isopeptide bond in SpaD   

**Figure 3:**
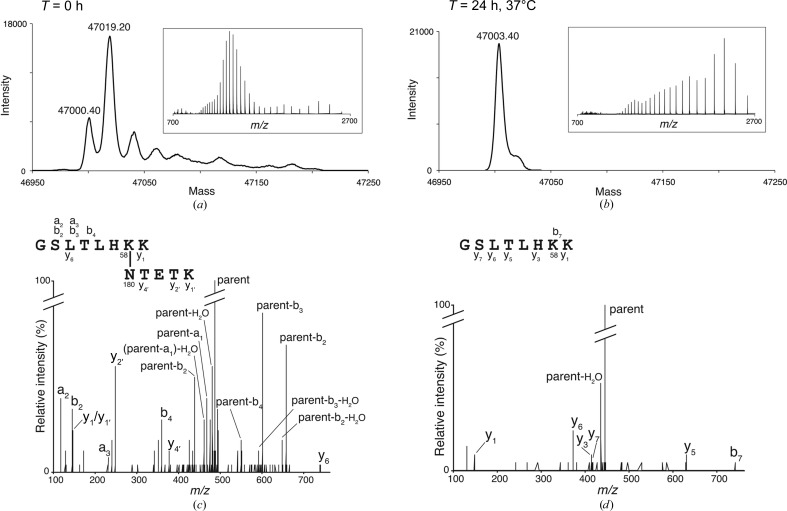
(*a*, *b*) Slow-forming isopeptide bond in SpaD. Mass spectrometry of freshly purified SpaD (*a*) revealed the presence of two protein species: one corresponding to SpaD with three isopeptide bonds (47 000.40 Da) and the other to SpaD with two isopeptide bonds (47 019.20 Da). When SpaD was incubated at 37°C for 24 h, the peak ratio changed and more of the three-isopeptide bond species were observed. The insets show charge envelopes before deconvolution. The observed charge envelope shifts to much higher *m*/*z* values when the third isopeptide bond forms as in (*b*), indicative of a tighter, more constrained protein structure with less surface area available for protonation. (*c*, *d*) LC-MS/MS of proteolytic products of SpaD containing peptides with and without the Lys58–Asn180 isopeptide-bond cross-link in domain D1. Fragmentation spectra of the parent ion at *m*/*z* 486.66^3+^ (*c*) indicate the presence of peptides cross-linked by the bond between SpaD Lys58 and Asn180, whereas the same sample also contained the ion at *m*/*z* 442.28^2+^ (*d*) showing a linear peptide containing Lys58. Daughter ions produced during MS/MS of these peptides are summarized in Tables 2 ▶ and 3 ▶.

Each of the three SpaD domains contains the conserved Lys–Asn–Asp/Glu triad characteristic of isopeptide bond-forming domains of this type, and all three bonds are fully formed in the crystal structure. However, mass spectrometry of freshly purified SpaD revealed the presence of two partially overlapping charge envelopes (Fig. 3 ▶). Deconvolution identified two protein species: one charge envelope corresponding to SpaD with three isopeptide bonds (47 000.4 Da) and the other to SpaD with two isopeptide bonds (47 019.2 Da).

The intensities of the deconvoluted peaks were noticeably different; the protein with two isopeptide bonds was more abundant than that with three, with an approximate ratio of 3:1. When SpaD was incubated at room temperature or 37°C, however, the peak ratio changed and more of the three-isopeptide-bond species were observed (Fig. 3 ▶). All three isopeptide bonds were fully formed in SpaD within 24 h of incubation at 37°C (and 72 h at room temperature). The protein crystals used in the structure determination were from freshly purified SpaD and the three isopeptide bonds seen in the structure must have fully formed during the time taken for crystallization (one to three weeks at 18°C).

The most likely candidate for the slow-forming bond is that in the D1 domain, by analogy with other three-domain and four-domain major pilins, such as GBS80, SpaA, BcpA and RrgB, whose N-domains either display no isopeptide bond or lack isopeptide-forming residues. Consistent with this, we detected a mixture of peptides with and without the Lys58–Asn180 isopeptide cross-link in domain D1 when the full-length protein was analysed by liquid chromatography-tandem mass spectrometry (LC-MS/MS) following a proteolytic digestion (Fig. 3 ▶, Table 2 ▶). In addition, when we produced a SpaD construct containing the D1 domain only (SpaD D1; residues 49–183), the ESI-TOF mass spectra showed two species, with the vast majority containing no isopeptide bond and a small minority containing a single isopeptide bond (Fig. 3 ▶). Interestingly, even after incubating the SpaD D1 protein for 46 h at 37°C, the ratio between species with the bond formed and unformed did not change (Fig. 3 ▶). Taken together, these results indicate that the D1 isopeptide bond forms only slowly in the recombinant protein, and that it forms much more readily in the presence of the rest of SpaD than it does in the isolated D1 domain lacking inter-domain contacts. We have attempted mass-spectral analysis of native SpaD pili purified from *C. diphtheriae*, but have not been successful and are unable to confirm whether the polymerized SpaD subunits in native pili have all three isopeptide bonds fully formed.

### 3.5. D1 domain: intermolecular isopeptide formation and freedom of motion   

**Figure 4:**
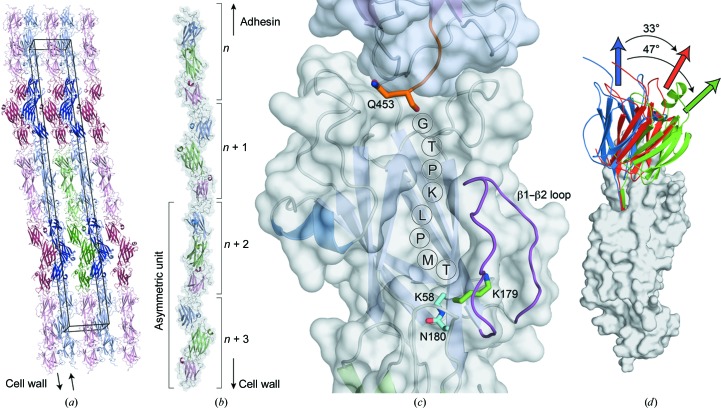
Crystal-packing implications for pilus assembly. (*a*) Crystal packing, showing pilus-like end-to-end stacking, with chain *A* shown in dark shades and chain *B* in light shades. The unit cell is indicated by a black box and a single asymmetric unit is shown in green. (*b*) A single pilus-like chain of molecules with domains coloured as per Fig. 1 ▶. Directionality is indicated along with the order of incorporation of pilin subunits. (*c*) Close-up of the interface between monomers. The C-terminal residue of the preceding monomer (Gln453, the last visible residue of molecule *B* in the crystal) is shown in orange, located at the entrance to the D1 groove, and would be followed in full-length SpaD by eight more residues, of which the last four belong to the LPMTG sortase-recognition motif. These missing residues and their predicted path are indicated. The intermolecular isopeptide bond-forming lysine is located at the base of this groove, in position to bond to the Thr of the sortase motif, and is coloured green. The β1–β2 loop (magenta) forms one side of this groove and covers the lysine residue. (*d*) Overlay of SpaD chain *A* (blue) and chain *B* (red) and SpaA (green) aligned based on D2 and D3 showing the varying orientations of D1. Domains D2 and D3 are shown as a light grey surface with D1 and the first strand of D2 as a smoothed cartoon. Arrows indicate the relative orientation of each D1, with the rotation angle of each domain around the hinge region (N-terminus of the first strand of D2) calculated by *DynDom* (Hayward & Berendsen, 1998 ▶).

Like other most major pilins, SpaD also contains within its N-terminal D1 domain the canonical YPKN pilin motif that contains the lysine residue, Lys179, used to form the sortase-mediated intermolecular isopeptide bond during polymerization (Fig. 2 ▶ *f*; Gaspar & Ton-That, 2006 ▶; Ton-That & Schneewind, 2003 ▶). The crystals of the full-length SpaD structure reveal end-to-end stacking of SpaD molecules (Figs. 4 ▶ *a* and 4 ▶ *b*), as is seen in many other major pilin crystal structures, and as would be expected in the authentic biological assembly following polymerization. In the crystal structure, the carboxyl-terminus of one molecule, which precedes the sortase-recognition motif in the construct analysed here, is located close to the D1 domain of the following molecule, near the start of a groove that leads to Lys179 from the YPKN motif, with the distance between these residues being consistent with the number of C-terminal residues missing from the model (Fig. 4 ▶ *c*).

A feature of the SpaD structure is that the D1 domain, as in other major pilins in which the N-terminal domains have been modelled, is more flexible than the other domains. This is seen in the variable orientation of D1 relative to D2/D3 in the two SpaD molecules, and in comparison with the homologous SpaA (Fig. 4 ▶ *d*). It is also reflected in the higher average *B* factors for the D1 domains relative to the D2/D3 domains (56.4 *versus* 45.8 Å^2^).

Of particular note, the 15-residue loop between strands β1 and β2 of the D1 domain is disordered in one molecule and could not be modelled owing to lack of electron density, while in the other molecule it is ordered enough to be modelled, albeit with high *B* factors. This loop extends over Lys179, restricting external access to it (Figs. 1 ▶ and 4 ▶ *c*). The equivalent loops in SpaA and FimP could not be modelled owing to poor density (Kang, Paterson, Gaspar *et al.*, 2009 ▶; Persson *et al.*, 2012 ▶), which is further evidence of the high degree of mobility of this feature. In the SpaD crystal structure the β1–β2 loop region is also adjacent to the C-terminus of the next SpaD molecule in the crystal, from which the sortase-recognition segment would extend.

## 4. Discussion   

SpaD, the subject of this report, is the major pilin protein that forms the polymeric backbone, or shaft, of one of the three types of pilus expressed by the human pathogen *C. diphtheriae*. These pilus assemblies have evolved to withstand severe physical and mechanical stresses while engaged with host cells during bacterial colonization. Their constituent pilin subunits are constructed from just two types of Ig-like domain, known as CnaB-type and CnaA-type, but these domains tend to be highly variable in their surface structures. Variations include added loops, helices and other features that presumably reflect their responses to immune pressure and to different host environments.

The SpaD structure most closely resembles that of SpaA, the major pilin of one of the two other pilus types from the same organism; they share the same domain structure and significant sequence and structural similarity, and are likely to have evolved from a common ancestor. SpaD and SpaA share several stabilizing features, including the isopeptide bonds in domains D2 and D3 and the disulfide bond in D3. SpaD has an additional isopeptide bond in its D1 domain, however, whereas in SpaA the residues corresponding to the isopeptide bond-forming Lys, Asn and Asp residues of SpaD are Ala, His and Gln, and no D1 isopeptide bond exists. The calcium-binding site of SpaD is also different in that it is in the C-­terminal domain D3 rather than in the middle domain as in SpaA. These differences may be relevant to the distinctive functions that these pili carry out, as is shown in their different host-cell preferences (Mandlik *et al.*, 2007 ▶).

The most intriguing feature of the SpaD structure is the slow-forming isopeptide bond in its N-terminal D1 domain. It has generally been assumed that the internal isopeptide bonds found in domains of this type form autocatalytically when the protein folds, when the reacting residues are brought into close proximity in a hydrophobic environment. In the present case, however, the recombinant SpaD protein was found to exist as two species: one with an isopeptide bond in its D1 domain and one without. Using mass spectrometry we could show, both for the isolated D1 domain protein and for full-length SpaD, that the isopeptide bond in the D1 domain forms slowly over time. There is a striking difference between the full-length and D1 proteins, however, in that the D1 bond becomes fully formed in the full-length protein, whereas that in the D1 construct remained mostly unformed even after prolonged incubation at 37°C. These results indicate that an energy barrier must be overcome that is much higher for the isolated domain. This conclusion fits with theoretical studies on isopeptide-bond formation in the *S. pyogenes* major pilin Spy0128, which show that domain–domain interactions can significantly influence bond formation (Hu *et al.*, 2011 ▶).

The slow-forming isopeptide bond in the SpaD D1 domain highlights an emerging consensus regarding the N-terminal domains of the major pilins of Gram-positive pili. Except in the case of Spy0128, these domains are both more flexible and more protease-sensitive than the other domains. Thus, the structures of the three-domain major pilins GBS80 and FimA (Vengadesan *et al.*, 2011 ▶; Mishra *et al.*, 2011 ▶) and of the four-domain major pilins BcpA and RrgB (Budzik *et al.*, 2009 ▶; Paterson & Baker, 2011 ▶; Spraggon *et al.*, 2010 ▶; Vetsch *et al.*, 2004 ▶) could be solved to high resolution only after their N-­terminal domains had been removed. Higher *B* factors and variable orientations for the N-terminal domains of all of the full-length major pilins for which structures are available, SpaA, SpaD (described here), RrgB and FimP, attest to the greater mobility of this domain, as does an NMR analysis of the D1 domain of RrgB (Gentile *et al.*, 2011 ▶). For full-length SpaD, the crystals were poorly ordered, probably owing to the flexibility of D1 coupled with the long unit-cell edge, and good diffraction was only achieved after cryocooling at high pressure.

The greater flexibility of the D1 domain is very likely to be linked to the sortase-mediated polymerization mechanism for Gram-positive pilus assembly. With the exception of Spy0128, the lysine that is linked by the sortase to the C-­terminus of the preceding pilin subunit is part of a conserved YPKN ‘pilin motif’ (Ton-That *et al.*, 2004 ▶). Importantly, the Lys residue (Lys179 in SpaD) is immediately adjacent to the asparagine (Asn180 in SpaD) that forms the internal D1 domain isopeptide bond. This generates a clear means by which formation of the internal isopeptide bond by Asn180 may be influenced by the sortase-mediated polymerization reaction involving Lys179; any movement of one residue is likely to affect the position of the other. Secondly, the YPKN motif is located on the final β-­strand of the D1 domain, very close to the D1–D2 interface, explaining why the presence or absence of the other domains can influence isopeptide-bond formation in D1, as we observe for SpaD.

Research on the major pilins BcpA and RrgB has provided both biochemical and structural evidence for a relationship between pilus assembly and internal isopeptide-bond formation. Recombinant BcpA has no isopeptide bond in its N-­terminal domain, but mass-spectral analysis shows that the bond is formed in the assembled pilus (Budzik *et al.*, 2009 ▶). Structural studies of full-length RrgB show how this may occur at the molecular level. In one RrgB crystal structure, no isopeptide bond was present in its N-terminal domain; the final β-strand of D1 contained a β-bulge at the site of the YPKN motif, displacing the Asn residue too far from the other isopeptide bond-forming residues (Paterson & Baker, 2011 ▶). In another crystal structure, however, for a construct that also included the C-terminal IPQTG sortase-recognition motif, it was found that the IPQTG peptide sits in a groove in the D1 domain of another RrgB molecule in the crystal, adjacent to the essential lysine, just as it would in polymerized pili (El Mortaji *et al.*, 2012 ▶). In this structure, the internal isopeptide bond is formed.

In our SpaD crystal structure the pilin molecules are stacked end-to-end, bringing the C-terminus of one molecule close to a groove in the N-terminal domain of the next (Fig. 4 ▶). This groove, between the β1–β2 loop and the main body of the domain, corresponds to the groove identified in RrgB; a similar groove is present in both SpaA (Kang, Paterson, Gaspar *et al.*, 2009 ▶) and FimP (Persson *et al.*, 2012 ▶). The essential lysine, Lys179 in SpaD, is in the floor of the groove and is mostly covered by the mobile β1–β2 loop. A similar β1–β2 loop is present in all major pilins for which full-length structures are available, except for Spy0128, which lacks an equivalent YPKN pilin motif. This β1–β2 loop flanks a similar groove in each case, but is usually disordered. In SpaD it is disordered in one molecule and ordered but with high *B* factors in the other. This flexibility may have a role in pilus polymerization, with the loop preventing unwanted inter­actions by covering the groove and then opening up to allow binding of the sortase-recognition segment of another molecule.

Our results showing a mixture of SpaD species, with the D1 internal isopeptide bond either formed or not formed, indicate that the bond in the N domain may not be fully formed in a SpaD monomer. An energy barrier clearly exists, possibly conformational in nature as shown for RrgB, and this can be overcome *in vitro* by warming. The low level of isopeptide-bond formation in the isolated D1 domain suggests a higher energy barrier in the absence of the other domains. The studies on RrgB and BcpA show that the energy barrier is also affected by docking of the sortase-recognition segment of another molecule and/or the sortase. We conclude that a flexible D1 domain, unconstrained by any internal cross-link, allows facile docking of the sortase-recognition segment, and possibly also the sortase, to enable formation of the intermolecular Lys–COO^−^ isopeptide-bond linkage. The stabilization of the D1 domain resulting from this protein–protein interaction then helps to overcome the energy barrier and allows the internal isopeptide bond to form, rigidifying the domain.

There are clearly differences from one major pilin to another, since the D1 bond forms more readily in SpaD than in RrgB in the absence of any protein–protein interaction. RrgB can also be readily polymerized *in vitro* by incubating with the pilus-polymerizing sortase, whereas SpaD could not when mixed with its cognate sortase (data not shown). Moreover SpaA, a close homologue of SpaD, does not contain isopeptide bond-forming residues in its N-terminal domain, whereas most of its homologues do (Kang, Paterson, Gaspar *et al.*, 2009 ▶). We speculate that this could endow specific functions or morphologies on SpaA pili, given that it has been shown recently that removing the bond in the N-terminal domain of BcpA prevents bundle formation of otherwise normal-looking BcpA pili (Hendrickx *et al.*, 2012 ▶).

Finally, it is important to note that the intermolecular isopeptide-bond linkages between successive subunits in the pilus polymer involve a lysine that is in most cases close to the boundary between the N-terminal domain and the following D2 domain. This means that the load-bearing ‘spine’ of the pilus does not pass through the N-terminal domain, but extends through the following domains, all of which are strengthened with internal isopeptide-bond cross-links. The role of the N-terminal domain may be to provide the site for sortase action, and possibly contribute to pilus morphology, making the presence or absence of an isopeptide cross-link less important.

## Supplementary Material

PDB reference: SpaD, 4hsq

PDB reference: 4hss

## Figures and Tables

**Table 1:** X-ray data-collection, phasing and refinement statistics Values in parentheses are for the outer shell.

	Full-length SpaD	SeMet SpaD_tryp_
Space group	*P*2_1_2_1_2_1_	*P*2_1_2_1_2_1_
Unit-cell parameters (Å, °)	*a* = 32.83, *b* = 56.79, *c* = 435.84, α = β = γ = 90.0	*a* = 35.99, *b* = 81.33, *c* = 92.37, α = β = γ = 90.0
Data collection		
Resolution range (Å)	29.06–2.50 (2.64–2.50)	19.86–1.87 (1.97–1.87)
Wavelength (Å)	0.91700	1.54178
Total reflections	143611 (21155)	922633 (115955)
Unique reflections	27956 (4066)	23176 (3255)
Multiplicity/anomalous multiplicity	5.1/3.0 (5.2/2.9)	39.8/21.2 (35.6/18.5)
Completeness (%)	95.2 (96.6)	99.6 (97.9)
Anomalous completeness (%)	87.7 (89.4)	99.5 (97.1)
〈*I*/σ(*I*)〉	10.9 (2.5)	21.2 (9.6)
*R*_merge_ (%)	10.2 (69.5)	8.7 (53.4)
Phasing statistics		
Resolution range (Å)		19.86–1.87
Figure of merit (acentric/centric)		0.05/0.16
Phasing power (anomalous)		0.31 [1.00 at 3.9 Å]
Sites		1 Se, 1 Ca, 2 S [disulfide]
Refinement statistics		
Resolution range (Å)	28.36–2.50 (2.59–2.50)	19.86–1.87 (1.95–1.87)
Total reflections (*R*_work_ + *R*_free_)	27924 (2902)	23113 (2733)
*R*_work_/*R*_free_ (%)	18.9/24.9 (23.8/36.9)	15.7/19.0 (18.4/23.0)
R.m.s. deviations		
Bond lengths (Å)	0.010	0.010
Angles (°)	1.23	1.14
Average *B* factor (Å^2^)	53.85	26.52
Protein atoms	6202	2110
Ions	2	1
Waters	281	346
Ramachandran plot by *MolProbity*		
Favoured/generously allowed (%)	97.2/2.5	98.1/1.9
Outliers (%)	0.0	0.0

**Table 2:** MS/MS of a linear peptide at *m*/*z* 442.28^2+^ containing Lys58 of SpaD

Observed *m*/*z* †	Charge	Calculated *m*/*z* ‡	Δ_obs−calc_	Proposed structure	Ion type
147.11	+1	147.11	0.00	K	y_1_
370.23	+2	370.25	−0.02	LTLHKK	y_6_
412.25	+1	412.26	−0.01	HKK	y_3_
413.77	+2	413.76	0.01	SLTLHKK	y_7_
433.30	+2	433.27	0.03	GSLTLHKK	Parent-H_2_O
442.28	+2	442.27	0.01	GSLTLHKK	Parent
626.45	+1	626.40	0.05	TLHKK	y_5_
737.39	+1	737.43	−0.04	GSLTLHK	b_7_

† Monoisotopic masses of observed ions.

‡ Calculated ions. Monoisotopic masses were calculated using the *Fragment Ion Calculator* (http://db.systemsbiology.net:8080/proteomicsToolkit/FragIonServlet.html).

**Table 3:** MS/MS of a peptide at *m*/*z* 486.66^3+^ containing the Lys58–Asn180 isopeptide bond of SpaD

Observed *m*/*z* †	Charge	Calculated *m*/*z* ‡	Δ_obs−calc_	Proposed structure	Ion type
117.08	+1	117.07	0.01	GS	a_2_
145.04	+1	145.06	−0.02	GS	b_2_
147.10	+1	147.11	−0.01	K	y_1_ or y_1′_
230.12	+1	230.20	−0.08	GSL	a_3_
248.16	+1	248.16	0.00	TK	y_2′_
359.20	+1	359.19	0.01	GSLT	b_4_
377.20	+1	377.20	0.00	TETK	y_4′_
438.63	+3	438.59	0.04	LTLHKK and NTETK (−NH_3_)§	Parent-b_2_
461.62	+3	461.60	0.02	SLTLHKK and NTETK (−NH_3_)§	Parent-G-H_2_O
467.63	+3	467.60	0.03	SLTLHKK and NTETK (−NH_3_)§	Parent-G
480.66	+3	480.60	0.06	GSLTLHKK and NTETK (−NH_3_)§	Parent-H_2_O
486.66	+3	486.60	0.06	GSLTLHKK and NTETK (−NH_3_)§	Parent
550.29	+2	550.31	−0.02	LHKK and NTETK (−NH_3_)§	Parent-b_4_
591.86	+2	591.83	0.03	TLHKK and NTETK (−NH_3_)§	Parent-b_3_-H_2_O
600.80	+2	600.84	−0.04	TLHKK and NTETK (−NH_3_)§	Parent-b_3_
657.29	+2	657.38	−0.09	LTLHKK and NTETK (−NH_3_)§	Parent-b_2_
739.32	+1	739.48	−0.16	LTLHKK	y_6_

† Monoisotopic masses of observed ions.

‡ Calculated ions. Monoisotopic masses were calculated using the *Fragment Ion Calculator* (http://db.systemsbiology.net:8080/proteomicsToolkit/FragIonServlet.html).

§ Loss of 17 Da from losing NH_3_ is shown in parentheses.
